# (*Z*)-1-(2,5-Dichloro-3-thien­yl)ethanone semicarbazone

**DOI:** 10.1107/S1600536809026567

**Published:** 2009-07-15

**Authors:** Hoong-Kun Fun, Ching Kheng Quah, A. M. Vijesh, Chitrakar Hegde, Arun M. Isloor

**Affiliations:** a X-ray Crystallography Unit, School of Physics, Universiti Sains Malaysia, 11800 USM, Penang, Malaysia; b SeQuent Scientific Limited, No. 120 A&B, Industrial Area, Baikampady, New Mangalore, Karnataka 575 011, India; c Department of Chemistry, NITTE Institute of Technology, Yelahanka, Bangalore 560 064, India; d Department of Chemistry, National Institute of Technology-Karnataka, Surathkal, Mangalore 575 025, India

## Abstract

The title mol­ecule, C_7_H_7_Cl_2_N_3_OS, is approximately planar [maximum deviation = 0.062 (1) Å]. Short inter­molecular distances between the centroids of the five-membered rings [3.5340 (8) Å] indicate the existence of π–π inter­actions. An inter­esting feature of the crystal structure is the presence of short intra­molecular Cl⋯N inter­actions [3.0015 (11) Å]. Mol­ecules are linked *via* pairs of inter­molecular N—H⋯O hydrogen bonds, generating *R*_2_^2^(8) ring motifs. Furthermore, N—H⋯O hydrogen bonds form *R*_2_^1^(7) ring motifs with C—H⋯O contacts, further consolidating the crystal structure. In the crystal, mol­ecules are linked by these inter­molecular inter­actions, forming chains along [001].

## Related literature

For the synthetic utility and applications of semicarbazone derivatives, see: Warren *et al.* (1977 ▶); Chandra & Gupta (2005 ▶); Jain *et al.* (2002 ▶); Pilgram (1978 ▶); Yogeeswari *et al.* (2004 ▶). For related structures, see: Fun *et al.* (2009*a* ▶,*b* ▶). For the preparation, see: Furniss *et al.* (1978 ▶). For hydrogen-bond motifs, see: Bernstein *et al.* (1995 ▶). For the stability of the temperature controller used for the data collection, see: Cosier & Glazer (1986 ▶).
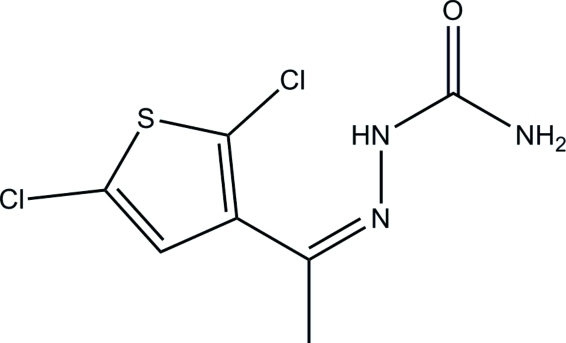


## Experimental

### 

#### Crystal data

C_7_H_7_Cl_2_N_3_OS
*M* *_r_* = 252.12
Monoclinic, 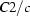

*a* = 13.0796 (2) Å
*b* = 10.4316 (2) Å
*c* = 14.4352 (2) Å
β = 94.599 (1)°
*V* = 1963.21 (6) Å^3^
*Z* = 8
Mo *K*α radiation
μ = 0.84 mm^−1^
*T* = 100 K
0.49 × 0.22 × 0.08 mm

#### Data collection

Bruker SMART APEXII CCD area-detector diffractometer
Absorption correction: multi-scan (**SADABS**; Bruker, 2005 ▶) *T*_min_ = 0.683, *T*_max_ = 0.934
16375 measured reflections
3742 independent reflections
3060 reflections with *I* > 2σ(*I*)
*R*_int_ = 0.029

#### Refinement

*R*[*F* ^2^ > 2σ(*F* ^2^)] = 0.034
*wR*(*F* ^2^) = 0.100
*S* = 1.13
3742 reflections
140 parameters
H atoms treated by a mixture of independent and constrained refinement
Δρ_max_ = 0.46 e Å^−3^
Δρ_min_ = −0.35 e Å^−3^

### 

Data collection: *APEX2* (Bruker, 2005 ▶); cell refinement: *SAINT* (Bruker, 2005 ▶); data reduction: *SAINT*; program(s) used to solve structure: *SHELXTL* (Sheldrick, 2008 ▶); program(s) used to refine structure: *SHELXTL*; molecular graphics: *SHELXTL*; software used to prepare material for publication: *SHELXTL* and *PLATON* (Spek, 2009 ▶).

## Supplementary Material

Crystal structure: contains datablocks global, I. DOI: 10.1107/S1600536809026567/tk2498sup1.cif

Structure factors: contains datablocks I. DOI: 10.1107/S1600536809026567/tk2498Isup2.hkl

Additional supplementary materials:  crystallographic information; 3D view; checkCIF report

## Figures and Tables

**Table 1:** Selected interatomic distance (Å)

*Cg*1⋯*Cg*1^i^	3.7188 (6)

Symmetry code: (i) 
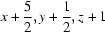
. *Cg*1 is the centroid of the S1/C3–C6 five-membered ring.

**Table 2:** Hydrogen-bond geometry (Å, °)

*D*—H⋯*A*	*D*—H	H⋯*A*	*D*⋯*A*	*D*—H⋯*A*
N1—H1*N*1⋯O1^ii^	0.86 (2)	2.02 (2)	2.8766 (15)	177.3 (19)
N2—H1*N*2⋯O1^iii^	0.863 (19)	2.035 (19)	2.8949 (14)	174.2 (17)
C7—H7*A*⋯O1^iii^	0.96	2.38	3.3370 (17)	176

Symmetry codes: (ii) 
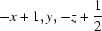
; (iii) 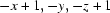.

**Fig. 1.**
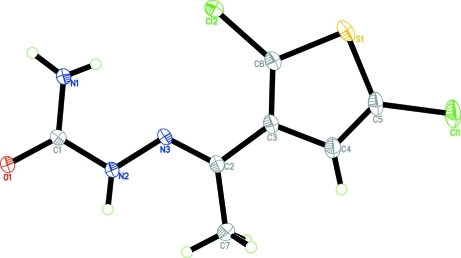
The molecular structure of (I), showing 50% probability displacement ellipsoids and the atom-numbering scheme.

**Fig. 2.**
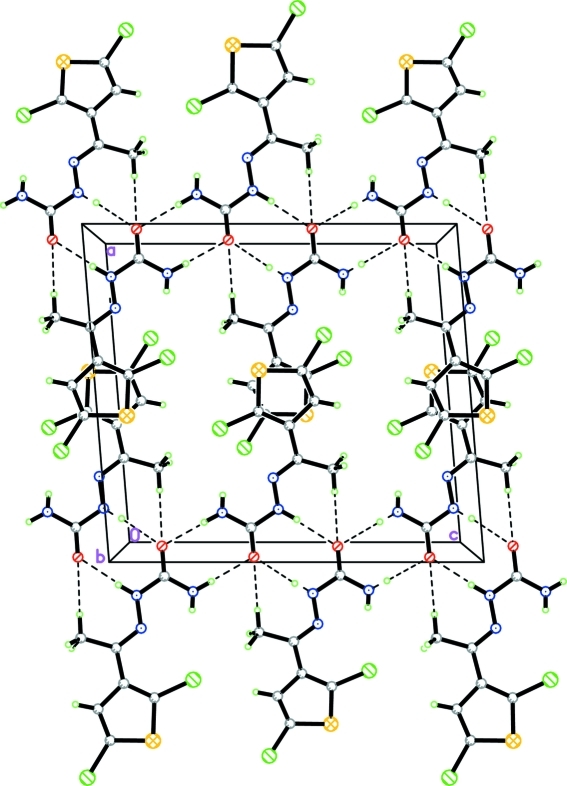
The crystal packing in (I), viewed along the b axis. The dashed lines indicate hydrogen bonds and C-H···O contacts.

## supplementary crystallographic information

### Comment

Semicarbazones find immense applications in the field of synthetic chemistry, such as medicinal chemistry (Warren *et al.*, 1977), organometallics (Chandra & Gupta, 2005), polymers (Jain *et al.*, 2002) and herbicides (Pilgram, 1978). Futher, 4-sulphamoylphenyl semicarbazones were found to possess anti-convulsant activity (Yogeeswari *et al.*, 2004). Herein, we report the crystal structure of the title semicarbazone (I).

The bond lengths and angles in (I), Fig. 1, are comparable to those observed in two closely related structures (Fun *et al.*, 2009*a*, b). The molecule is approximately planar, with an r.m.s. deviation of 0.062 (1) Å for atom O1. The short intramolecular distances between the centroids of five-membered rings [3.5340 (8) Å] prove existence of π–π interactions (Table 1). The interesting feature of the crystal structure is the short intermolecular Cl···N interactions [3.0015 (11) Å]".

The molecules are linked *via* pairs of intermolecular N1—H1N1···O1 and N2—H1N2···O1 (Table 2) hydrogen bonds to generate *R*_2_^2^(8) ring motifs (Bernstein *et al.*, 1995) (Fig. 2). Furthermore, N2—H1N2···O1 hydrogen bonds form *R*_2_^1^(7) ring motifs with C7—H7A···O1 contacts to further consolidate the crystal structure. The molecules are linked by these intermolecular interactions to form 1-D chains along the [0 0 1] direction.

### Experimental

Semicarbazide hydrochloride (1.84 g, 16.5 mmol) and freshly recrystallized sodium acetate (1.64 g, 20.0 mmol) was dissolved in water (15 ml) according to a literature procedure (Furniss *et al.*, 1978). The reaction mixture was stirred at room temperature for 10 minutes. To this, 2,5-dichloro-3-acetylthiophene (3.0 g, 15.4 mmol) in  ethanol (15 ml) was added and stirred well for 6 h. The separated semicarbazone was filtered, washed with chilled water and recrystallized from an ethanol/dimethylformamide mixture. Yield: 3.19 g, 82.22%. *M.p.* 491–493 K.

### Refinement

N-bound H atoms were located in a difference Fourier map and were allowed to refine freely, see Table 2 for distances. All the other H atoms were placed in calculated positions, with C–H = 0.93 or 0.96 Å, and refined using a riding model with *U*_iso_(H) = 1.2 or 1.5 *U*_eq_(C). A rotating-group model was applied for the methyl group.

### Crystal data

**Table:**

C_7_H_7_Cl_2_N_3_OS	*F*(000) = 1024
*M**_r_* = 252.12	*D*_x_ = 1.706 Mg m^−^^3^
Monoclinic, *C*2/*c*	Mo *K*α radiation, λ = 0.71073 Å
Hall symbol: -C 2yc	Cell parameters from 6313 reflections
*a* = 13.0796 (2) Å	θ = 2.5–33.2°
*b* = 10.4316 (2) Å	µ = 0.84 mm^−^^1^
*c* = 14.4352 (2) Å	*T* = 100 K
β = 94.599 (1)°	Plate, colourless
*V* = 1963.21 (6) Å^3^	0.49 × 0.22 × 0.08 mm
*Z* = 8	

### Data collection

**Table:**

Bruker SMART APEXII CCD area-detector diffractometer	3742 independent reflections
Radiation source: fine-focus sealed tube	3060 reflections with *I* > 2σ(*I*)
graphite	*R*_int_ = 0.029
φ and ω scans	θ_max_ = 33.2°, θ_min_ = 2.5°
Absorption correction: multi-scan (*SADABS*; Bruker, 2005)	*h* = −20→19
*T*_min_ = 0.683, *T*_max_ = 0.934	*k* = −16→15
16375 measured reflections	*l* = −22→22

### Refinement

**Table:**

Refinement on *F*^2^	Primary atom site location: structure-invariant direct methods
Least-squares matrix: full	Secondary atom site location: difference Fourier map
*R*[*F*^2^ > 2σ(*F*^2^)] = 0.034	Hydrogen site location: inferred from neighbouring sites
*wR*(*F*^2^) = 0.100	H atoms treated by a mixture of independent and constrained refinement
*S* = 1.13	*w* = 1/[σ^2^(*F*_o_^2^) + (0.0516*P*)^2^ + 0.6332*P*] where *P* = (*F*_o_^2^ + 2*F*_c_^2^)/3
3742 reflections	(Δ/σ)_max_ = 0.002
140 parameters	Δρ_max_ = 0.46 e Å^−^^3^
0 restraints	Δρ_min_ = −0.35 e Å^−^^3^

### Special details

**Table:**

Experimental. The crystal was placed in the cold stream of an Oxford Cyrosystems Cobra open-flow nitrogen cryostat (Cosier & Glazer, 1986) operating at 100.0 (1) K.
Geometry. All e.s.d.'s (except the e.s.d. in the dihedral angle between two l.s. planes) are estimated using the full covariance matrix. The cell e.s.d.'s are taken into account individually in the estimation of e.s.d.'s in distances, angles and torsion angles; correlations between e.s.d.'s in cell parameters are only used when they are defined by crystal symmetry. An approximate (isotropic) treatment of cell e.s.d.'s is used for estimating e.s.d.'s involving l.s. planes.
Refinement. Refinement of *F*^2^ against ALL reflections. The weighted *R*-factor *wR* and goodness of fit *S* are based on *F*^2^, conventional *R*-factors *R* are based on *F*, with *F* set to zero for negative *F*^2^. The threshold expression of *F*^2^ > σ(*F*^2^) is used only for calculating *R*-factors(gt) *etc*. and is not relevant to the choice of reflections for refinement. *R*-factors based on *F*^2^ are statistically about twice as large as those based on *F*, and *R*- factors based on ALL data will be even larger.

### Fractional atomic coordinates and isotropic or equivalent isotropic displacement parameters (Å^2^)

**Table:**

	*x*	*y*	*z*	*U*_iso_*/*U*_eq_	
Cl1	1.17736 (3)	0.23231 (4)	0.62567 (3)	0.02794 (10)	
Cl2	0.88955 (3)	0.12728 (4)	0.31571 (2)	0.02421 (10)	
S1	1.06944 (2)	0.19596 (3)	0.43833 (3)	0.01940 (9)	
O1	0.48318 (7)	−0.00827 (9)	0.37962 (6)	0.01622 (18)	
N1	0.62401 (10)	0.02718 (15)	0.30045 (8)	0.0247 (3)	
N2	0.63508 (8)	0.03704 (11)	0.46052 (7)	0.0150 (2)	
N3	0.73470 (8)	0.07286 (10)	0.45321 (7)	0.0146 (2)	
C1	0.57608 (9)	0.01731 (12)	0.37865 (8)	0.0147 (2)	
C2	0.79348 (9)	0.09889 (12)	0.52643 (9)	0.0141 (2)	
C3	0.89959 (10)	0.13686 (12)	0.51073 (9)	0.0150 (2)	
C4	0.97547 (10)	0.16495 (13)	0.58619 (9)	0.0183 (2)	
H4A	0.9621	0.1617	0.6485	0.022*	
C5	1.06803 (10)	0.19643 (13)	0.55714 (10)	0.0194 (3)	
C6	0.94260 (10)	0.15031 (13)	0.42725 (9)	0.0167 (2)	
C7	0.76299 (11)	0.09363 (16)	0.62397 (9)	0.0232 (3)	
H7A	0.6913	0.0734	0.6234	0.035*	
H7B	0.7757	0.1753	0.6533	0.035*	
H7C	0.8024	0.0288	0.6579	0.035*	
H1N1	0.5931 (16)	0.0142 (19)	0.2466 (16)	0.036 (5)*	
H2N1	0.6797 (17)	0.0442 (19)	0.3063 (14)	0.030 (5)*	
H1N2	0.6027 (14)	0.0324 (17)	0.5102 (13)	0.022 (4)*	

### Atomic displacement parameters (Å^2^)

**Table:**

	*U*^11^	*U*^22^	*U*^33^	*U*^12^	*U*^13^	*U*^23^
Cl1	0.01347 (16)	0.0327 (2)	0.0365 (2)	−0.00378 (13)	−0.00478 (14)	−0.00720 (15)
Cl2	0.01543 (15)	0.0411 (2)	0.01659 (15)	−0.00354 (13)	0.00432 (11)	0.00003 (12)
S1	0.01115 (15)	0.02034 (16)	0.02707 (17)	−0.00210 (11)	0.00391 (12)	0.00104 (12)
O1	0.0101 (4)	0.0251 (5)	0.0135 (4)	−0.0015 (4)	0.0005 (3)	0.0007 (3)
N1	0.0116 (5)	0.0502 (8)	0.0124 (5)	−0.0061 (5)	0.0008 (4)	−0.0013 (5)
N2	0.0102 (4)	0.0224 (5)	0.0122 (4)	−0.0034 (4)	0.0008 (4)	−0.0002 (4)
N3	0.0095 (4)	0.0199 (5)	0.0148 (4)	−0.0027 (4)	0.0018 (4)	−0.0003 (4)
C1	0.0121 (5)	0.0185 (6)	0.0136 (5)	−0.0006 (4)	0.0020 (4)	−0.0001 (4)
C2	0.0113 (5)	0.0156 (5)	0.0152 (5)	−0.0010 (4)	0.0010 (4)	−0.0004 (4)
C3	0.0113 (5)	0.0154 (5)	0.0182 (5)	−0.0006 (4)	0.0006 (4)	−0.0008 (4)
C4	0.0137 (6)	0.0199 (6)	0.0208 (6)	−0.0011 (5)	−0.0010 (5)	−0.0029 (5)
C5	0.0121 (5)	0.0195 (6)	0.0261 (6)	−0.0011 (5)	−0.0020 (5)	−0.0041 (5)
C6	0.0117 (5)	0.0192 (6)	0.0192 (6)	−0.0010 (5)	0.0016 (4)	0.0000 (4)
C7	0.0164 (6)	0.0390 (8)	0.0140 (5)	−0.0029 (6)	0.0005 (5)	−0.0002 (5)

### Geometric parameters (Å, °)

**Table:**

Cl1—C5	1.7137 (14)	N3—C2	1.2851 (16)
Cl2—C6	1.7182 (13)	C2—C3	1.4782 (18)
S1—C5	1.7167 (15)	C2—C7	1.4949 (18)
S1—C6	1.7211 (13)	C3—C6	1.3773 (19)
O1—C1	1.2452 (15)	C3—C4	1.4436 (18)
N1—C1	1.3384 (17)	C4—C5	1.3530 (19)
N1—H1N1	0.86 (2)	C4—H4A	0.9300
N1—H2N1	0.75 (2)	C7—H7A	0.9600
N2—N3	1.3677 (15)	C7—H7B	0.9600
N2—C1	1.3741 (16)	C7—H7C	0.9600
N2—H1N2	0.863 (19)		
			
Cg1···Cg1^i^	3.7188 (6)	Cl2···N3	3.0015 (11)
			
C5—S1—C6	90.35 (6)	C4—C3—C2	122.41 (12)
C1—N1—H1N1	122.3 (14)	C5—C4—C3	113.17 (13)
C1—N1—H2N1	116.2 (16)	C5—C4—H4A	123.4
H1N1—N1—H2N1	122 (2)	C3—C4—H4A	123.4
N3—N2—C1	116.57 (10)	C4—C5—Cl1	126.87 (12)
N3—N2—H1N2	127.8 (12)	C4—C5—S1	112.98 (10)
C1—N2—H1N2	115.3 (12)	Cl1—C5—S1	120.14 (8)
C2—N3—N2	120.34 (11)	C3—C6—Cl2	130.02 (10)
O1—C1—N1	123.33 (12)	C3—C6—S1	113.90 (10)
O1—C1—N2	120.24 (11)	Cl2—C6—S1	116.08 (8)
N1—C1—N2	116.44 (11)	C2—C7—H7A	109.5
N3—C2—C3	115.96 (11)	C2—C7—H7B	109.5
N3—C2—C7	125.43 (11)	H7A—C7—H7B	109.5
C3—C2—C7	118.61 (11)	C2—C7—H7C	109.5
C6—C3—C4	109.59 (11)	H7A—C7—H7C	109.5
C6—C3—C2	128.00 (12)	H7B—C7—H7C	109.5
			
C1—N2—N3—C2	176.77 (12)	C3—C4—C5—Cl1	−178.46 (10)
N3—N2—C1—O1	−175.87 (11)	C3—C4—C5—S1	0.65 (16)
N3—N2—C1—N1	4.15 (18)	C6—S1—C5—C4	−0.70 (11)
N2—N3—C2—C3	−179.86 (11)	C6—S1—C5—Cl1	178.47 (9)
N2—N3—C2—C7	0.1 (2)	C4—C3—C6—Cl2	179.02 (11)
N3—C2—C3—C6	1.5 (2)	C2—C3—C6—Cl2	−0.5 (2)
C7—C2—C3—C6	−178.55 (13)	C4—C3—C6—S1	−0.35 (15)
N3—C2—C3—C4	−177.95 (12)	C2—C3—C6—S1	−179.82 (10)
C7—C2—C3—C4	2.03 (19)	C5—S1—C6—C3	0.60 (11)
C6—C3—C4—C5	−0.19 (17)	C5—S1—C6—Cl2	−178.86 (9)
C2—C3—C4—C5	179.32 (12)		

Symmetry codes: (i) *x*+5/2, *y*+1/2, *z*+1.

### Hydrogen-bond geometry (Å, °)

**Table:**

*D*—H···*A*	*D*—H	H···*A*	*D*···*A*	*D*—H···*A*
N1—H1N1···O1^ii^	0.86 (2)	2.02 (2)	2.8766 (15)	177.3 (19)
N2—H1N2···O1^iii^	0.863 (19)	2.035 (19)	2.8949 (14)	174.2 (17)
C7—H7A···O1^iii^	0.96	2.38	3.3370 (17)	176

Symmetry codes: (ii) −*x*+1, *y*, −*z*+1/2; (iii) −*x*+1, −*y*, −*z*+1.
